# Algorithm-guided treatment for major depressive disorder versus treatment as usual: a systematic review

**DOI:** 10.3389/fpsyt.2026.1765024

**Published:** 2026-03-25

**Authors:** Deni Rkman, René Ernst Nielsen, Rasmus W. Licht, Klaus Martiny, Philipp Ritter, Marton Asztalos

**Affiliations:** 1 Department of Psychiatry, Aalborg University Hospital, Aalborg, Denmark; 2 Department of Clinical Medicine, Aalborg University, Aalborg, Denmark; 3 Department of Clinical Medicine, University of Copenhagen, Copenhagen, Denmark; 4 Copenhagen Affective Disorder Research Center (CADIC), Psychiatric Center, Copenhagen, Denmark; 5 Department of Psychiatry and Psychotherapy, University Hospital Carl Gustav Carus, Technical University Dresden, Dresden, Germany; 6 Institute of Psychiatry, Psychology and Neuroscience, King’s College London, London, United Kingdom

**Keywords:** algorithm-guided treatment, treatment as usual, depression, major depressive disorder, measurement-based care, systematic review

## Abstract

**Introduction:**

A substantial proportion of patients with major depressive disorder do not remit after the initial pharmacological treatment, and a major obstacle is that progression to subsequent treatment steps often occurs too slowly, highlighting the need for more structured and effective therapeutic strategies. Algorithm-guided treatments (AGTs) provide a systematic, stepwise framework for clinical decision-making, potentially improving acute treatment outcomes compared to treatment as usual (TAU).

**Methods:**

This systematic review, conducted according to PRISMA 2020 guidelines, evaluated randomized controlled trials (RCTs) comparing AGTs to TAU in adult patients with major depressive disorder. Databases searched included PubMed, Scopus, Embase, PsychInfo, and the Cochrane Library up to June 2025. Trials investigating adults diagnosed with major depressive disorder, utilizing clinician-rated depression scales, and with a trial duration of four weeks or more were included.

**Results:**

Seven RCTs met the criteria, encompassing over 3,500 participants. Most studies demonstrated superior outcomes in participants allocated to AGT compared to TAU, including significantly shorter time to remission, a higher proportion of patients achieving remission and response, as well as better adherence to treatment protocols. Some studies found marginal or nonsignificant differences between interventions in some of the outcomes, particularly those involving comorbid populations.

**Discussion:**

These findings suggest that implementing structured, algorithm-based treatment strategies can improve the quality and efficacy of care for patients diagnosed with major depressive disorder, supporting their wider integration into clinical practice.

## 1 Introduction

Depressive disorders are one of the leading contributors to the global burden of disease and were ranked second and thirteenth among the top 25 leading causes of years lived with disabilities (YLDs) and disability-adjusted life-years (DALYs), respectively (1). Even with a wide range of therapy choices, treatment refractory depression still presents a significant challenge. It has been shown that about 33% of patients with major depressive disorder (MDD) achieved remission after the first treatment step (2). Among those who did not remit, an additional 20% remitted after the second step, bringing the cumulative proportion of patients achieving remission to approximately 53%. A further 10% of patients remitted after the third step, and 7% after the fourth, resulting in a total cumulative proportion of patients achieving remission of around 70% after four successive treatment trials (2). Each treatment step lasts approximately 9–10 weeks, with remission typically occurring within the first 6 weeks. Consequently, patients progressing through four successive treatment trials may remain in active treatment for approximately 9 months.

These findings highlight that with each additional failed trial, the likelihood of remission declines substantially, and that patients who do not respond early are increasingly at risk of developing chronic or treatment-resistant depression. Further, with an increasing number of treatment attempts, an increase in discontinuation due to side effects was observed (3). This underlines the importance of systematic early monitoring of symptom change during an adequate treatment trial (typically 4 weeks at a recognized therapeutic dose), while also acknowledging evidence from meta-analyses of RCTs that lack of early improvement within the first 1–2 weeks predicts a lower likelihood of later response/remission (4). Thus, “timely adjustment” should be understood as measurement-informed reassessment and planning during the adequate trial, with treatment changes considered when there is minimal or no improvement after an adequate duration and dose. Despite these well-described patterns, treatment modification in routine clinical practice is often delayed or does not occur even when patients fail to show early symptom improvement (5). Antidepressant treatments may be continued for prolonged periods without systematic evaluation or timely escalation, switching, or augmentation, thereby prolonging exposure to ineffective interventions. Such delays in treatment adjustment may contribute to the accumulation of unsuccessful treatment trials and increase the risk of poor long-term outcomes.

One approach to systematically addressing these challenges is algorithm-guided treatment (AGT), in which predefined, evidence-based decision rules structure sequential treatment choices. The essential elements of AGTs are which treatments to use, how to implement each treatment and in what order to implement the different treatments (6). Furthermore, AGTs also define critical decision points where symptom measurements result in continuation or adjustment of current treatment strategy based on preset “if–then rules.” Developing a systematic and diligent approach could enhance patient outcomes, reduce treatment resistance, increase the quality of care, and decrease direct and indirect costs of healthcare (7). By using AGTs, clinicians are provided with a clear framework for decision-making, concurrently enhancing patient satisfaction and overall quality of life (8). AGT overlaps conceptually with a guideline implementation and measurement-based care (MBC). While MBC is defined as a clinical approach in which outcome measures are routinely collected and used to guide treatment decisions, AGT additionally incorporates a prespecified, sequenced, stepwise approach to treatment recommendations (9). Previous systematic reviews have examined the implementation of psychiatric guidelines and treatment algorithms, often including algorithmic decision support as one element within wider implementation strategies (10, 11). In these studies, guideline implementation primarily aims to improve adherence to evidence-based recommendations, while MBC focuses on systematic symptom monitoring to inform clinical judgment. In contrast, AGT more explicitly integrates symptom measurements into prespecified, sequenced treatment pathways guided by decision rules.

Several studies have been conducted investigating the role of systematic, stepwise, drug treatment protocols and regimes in individuals diagnosed with depression. Some of these studies, such as Sequenced Treatment Alternatives to Relieve Depression (STAR*D) (2), compared different treatment strategies with each other, while the German Algorithm Project 3 Trial (GAP3) compared AGTs with treatment as usual (12). The Texas Medication Algorithm Project also utilized treatment as usual as comparator in a cluster randomized design (13).

In this systematic review, we aim to evaluate the clinical effectiveness of AGT compared to TAU in adults diagnosed with major depressive disorder. TAU is defined as the locally accepted treatment practice for depression and reflects standard clinical care (14). Only randomized controlled trials (RCTs) that applied structured, stepwise pharmacological algorithms and reported clinician-rated outcomes were included. By synthesizing the available evidence, our goal is to assess whether AGT improves a proportion of patients achieving remission, response rates, time to remission, and overall treatment adherence as compared to TAU.

## 2 Materials and methods

### 2.1 Study design

Our review was conducted following the Preferred Reporting Items for Systematic Reviews and Meta-Analyses (PRISMA) statement (15). The completed PRISMA 2020 checklist is provided in **Supplementary Material 1**.

### 2.2 Search strategy and data sources

PubMed, Scopus, PsycINFO, Embase and the Cochrane Library Databases were systematically and independently searched by DR and last updated on June 30, 2025. Search terms included: (algorithm-based treatment) OR (algorithm-guided treatment) AND (treatment as usual) AND (depressi*). The original term search is presented in **Supplementary Material 2**. Relevant reviews were hand-searched for additional relevant studies. Afterwards two of the authors, DR and MA, independently screened the titles and abstracts of relevant publications and then read the relevant full texts for eligibility. After extracting the dataset, Rayyan was used to assist in the removal of duplicate references (16). Any disagreement was resolved by a discussion between DR and MA or subsequently by consulting REN. If it was necessary, additional information was obtained by contacting corresponding authors (or other authors, if the corresponding author did not respond). Although this review was not prospectively registered (e.g., PROSPERO), the methods were prespecified in a written protocol prior to database searching and screening. No deviations from the protocol occurred. The protocol is provided as **Supplementary Material 3**.

### 2.3 Data extraction

Outcomes were extracted by DR and MA on a pre-specified extraction form. In addition to the title, authors, publication year, and patient demographics, we extracted key study characteristics including setting, sample size, intervention and comparator, treatment duration, clinician-rated scales used, and the primary and secondary outcomes reported. Extracted outcome data is presented in the text and in **Table 1**.

**Table 1:** Study and population characteristics.

Article #	Title	Author and year	N (M/F)	Mean age	Population	Study settings	Primary outcome(s)
1	Evaluating the efficacy and moderators of algorithm-guided antidepressant treatments of major depressive disorder	Zhang H et al., 2022 (22)	987 (333/640)*	38.2	Adults aged 18–70 years with major depressive disorder and moderate to severe depressive symptoms	Multicentre psychiatric outpatient clinics across eight hospitals	Change in HAMD-based remission (HAMD-17 total score ≤ 7) or QIDS-based remission (QIDS-SR16 total score ≤ 5) from baseline to end of 6 weeks acute treatment phase
2	The clinical effectiveness of using a predictive algorithm to guide antidepressant treatment in primary care (PReDicT): an open-label, randomised controlled trial	Browning M et al., 2021 (23)	913 (346/567)	39	Adults aged 18–70 years with a depressive episode initiating antidepressant treatment	Multicentre primary care clinics across five countries	Response of depression symptoms (≥50% reduction in baseline score of the QIDS-SR-16 scale, at week 8)
3	How Effective Is Algorithm-Guided Treatment for Depressed Inpatients? Results from the Randomized Controlled Multicenter German Algorithm Project 3 Trial	Adli M et al., 2017 (12)	429 (157/272)	44.2	Adults aged 18–70 years with a current major depressive episode	Multicentre psychiatric inpatient units across 10 hospitals	Time to remission (HAMD-21 score ≤9)
4	Measurement-Based Care Versus Standard Care for Major Depression: A Randomized Controlled Trial With Blind Raters	Tong Guo et al., 2015 (18)	120 (43/77)	41.1	Adults aged 18–65 years with a major depressive disorder, with moderate to severe depressive symptoms	Single-centre psychiatric outpatient clinic	Time to remission (HAMD-17 score ≤7) and time response (decrease of ≥50% in HAMD-17 score)
5	Collaborative care management of major depression among low-income, predominantly Hispanic subjects with diabetes	Kathleen Ell et al., 2010 (19)	387 (69/318)	-*	Adults aged 18 years and older, with major depression and comorbid diabetes	Multicenter study conducted across two safety-net primary care clinics	Response of depression symptoms (≥50% reduction in Symptom Checklist-20 depression score)
6	Reducing Suicidal Ideation and Depression in Older Primary Care Patients: 24-Month Outcomes of the PROSPECT Study	Alexopoulos, George S et al., 2009 (21)	599 (170/429)	-*	Adults aged 60 years and older with depression treated in primary care	Multicentre primary care clinics across 20 practices	Reduction in suicidal ideation measured on the Scale for Suicide Ideation and rates of treatment response (decrease of ≥50% in HAMD-17 score from baseline) and remission (HAMD-17 score under 7)
7	Efficacy of an algorithm-guided treatment compared with treatment as usual: a randomized, controlled study of inpatients with depression	Bauer M et al., 2009 (20)	148 (60/88)	48.2	Adults aged 18 and older with major depressive episode	Single-center psychiatric inpatient unit	Time to remission (Bech-Rafaelsen Melancholia Scale score of ≤7)

*Due to missing date on some characteristics, the numbers of patients do not always add up.

### 2.4 Inclusions and exclusions criteria

Only randomized trials evaluating AGT to TAU were included. TAU is defined as the locally accepted treatment practice for depression and reflects standard clinical care. It was not required to use a specific definition of TAU. Inclusion criteria were: (i) adult patients diagnosed with major depressive disorder were to be included; (ii) co-morbid disorders, except those mentioned as exclusion criteria, were allowed; (iii) trial duration was four weeks or more; (iv) change in symptoms over trial between interventions was measured on a clinician-rated scale; (v) publications were reported in English. As defined in the Introduction (6), all included studies were classified as AGT, as they each incorporated the core elements of AGT to varying degrees. As summarized in **Table 2**, these elements include prespecified treatment options, structured treatment sequencing, and symptom-triggered decision rules applied at predefined or scheduled decision points. Exclusion criteria were: (i) trials published only as conference abstracts; (ii) patients diagnosed with bipolar disorder, organic affective disorders, schizophrenia; (iii) studies including patients with substance misuse or severe comorbid physical disease or terminal cancer.

**Table 2:** Algorithm characteristics.

Article # and title	Study duration	Frequency of assessments	Core components	Decision points	Implementation modality
1. Evaluating the efficacy and moderators of algorithm-guided antidepressant treatments of major depressive disorder	6-12-weeks, 6-months posttreatment follow-up	2, 4, 6, 8, 12 weeks, 6-month follow-up	Predefined, stepwise pharmacological treatment algorithm with measurement-based guidance	Predefined decision points at each scheduled visit based on symptom response and tolerability	If symptom severity at predefined assessment intervals met response thresholds, algorithm defined treatment recommendations were generated, which clinicians implemented within the predefined stepwise treatment plan
2. The clinical effectiveness of using a predictive algorithm to guide antidepressant treatment in primary care (PReDicT): an open-label, randomised controlled trial	48 weeks	Baseline, weeks 1 and 2, followed by regular follow-up assessments up to week 48	Computerized decision-support system predicting early antidepressant response to guide initial treatment selection	Two predefined decision points based on early symptom change	Algorithm generated treatment-modification recommendations at weeks 1 and 2, subsequent care followed routine clinical practice
3. How Effective Is Algorithm-Guided Treatment for Depressed Inpatients? Results from the Randomized Controlled Multicenter German Algorithm Project 3 Trial	20 weeks	Every 4 weeks, with additional 2-week reassessments for remission confirmation or step prolongation when indicated	Algorithm-guided, standardized stepwise drug treatment regimen, incorporating predefined antidepressant strategies and systematic clinician-rated symptom monitoring	Predefined algorithm decision points determine treatment according to prespecified if–then rules	Algorithm-driven implementation using predefined stepwise treatment pathways, with clinicians applying algorithm-specified treatment changes based on symptom response at scheduled decision points
4. Measurement-Based Care Versus Standard Care for Major Depression: A Randomized Controlled Trial With Blind Raters	24 weeks	baseline, weeks 2, 4, 8, 12, and 24	Measurement-based treatment algorithm with predefined dosing and escalation steps informed by symptom and side-effect ratings	Predefined decision points based on symptom change	Treatment followed a measurement-based algorithm with predefined steps, in which symptom and side-effect ratings at each visit guided if–then decisions on dose escalation, continuation, or medication change according to a prespecified schedule
5. Collaborative care management of major depression among low-income, predominantly Hispanic subjects with diabetes	18 months	Regular symptom assessments during initiation and adjustment of therapy (weeks 1-12), followed by monthly symptom monitoring during the maintenance phase	Treatment followed a stepped-care model in which medication and/or psychotherapy were adjusted based on symptom outcomes	Scheduled assessment points informing treatment recommendations within the stepped-care model	Treatment initiated based on patient preference. Partial or nonresponse prompted switching or augmentation, while responders entered maintenance monitoring and nonresponders were considered for further augmentation or further referral
6. Reducing Suicidal Ideation and Depression in Older Primary Care Patients: 24-Month Outcomes of the PROSPECT Study	2 years	4, 8, 12, and 24 months	Algorithm-guided collaborative care intervention incorporating a structured, guideline-based treatment algorithm and systematic clinician-rated symptom monitoring	Predefined decision points based on clinician-rated symptoms severity and tolerability, guiding further treatment steps	Algorithm-informed, collaborative implementation where care managers monitored symptoms and applied the stepped-care algorithm, communicated treatment recommendations to primary care clinicians, while final treatment implementation remained clinician-led
7. Efficacy of an algorithm-guided treatment compared with treatment as usual: a randomized, controlled study of inpatients with depression	Up to approximately 20 weeks, depending on progression through the stepwise treatment algorithm	Intervals ranging from 3 to 28 days across 10 steps	Algorithm-guided standardized stepwise treatment regimen, incorporating predefined strategies and systematic symptom monitoring	Predefined algorithm decision points based on symptoms severity determined treatment progression according to prespecified if–then rules	Algorithm-driven implementation, with treating clinicians following a predefined stepwise treatment sequence guided by clinician-rated symptom thresholds at scheduled decision points

### 2.5 Quality assessment

Only randomized trials, in which all inclusion criteria were fulfilled, and no exclusion criteria were fulfilled, were included in the review.

### 2.6 Risk of Bias

Across the included trials, the overall risk of bias for the primary outcome was judged to have some concerns (**Table 3**). This was driven predominantly by the open-label nature of the interventions and the complexity of algorithm-guided and collaborative-care strategies, which make deviations from intended interventions and performance effects difficult to fully exclude. Furthermore, in studies where AGT and TAU were delivered within the same clinical setting, algorithm-based treatment may have influenced the management of patients allocated to TAU. Consequently, between-group differences may have been attenuated, resulting in smaller observed differences between groups and a potential underestimation of the effect of algorithm-guided treatment. In most trials, primary outcomes were clinician-rated or interviewer-assessed symptom measures, and blinding of outcome assessors was either not feasible or incompletely reported, leading to concerns regarding measurement bias. In contrast, randomization procedures were generally adequate, attrition was low or comparable between groups in most studies, and there was no clear evidence of selective outcome reporting.

**Table 3:** Risk of bias assessment (Cochrane RoB 2).

Study	Randomization process	Deviations from intended interventions	Missing outcome data	Measurement of the outcome	Selection of the reported result	Overall risk of bias
Adli et al., 2017 (GAP-3) (12)	Low	Some concerns	Some concerns	Some concerns	Low	Some concerns
Guo et al., 2015 (18)	Low	Some concerns	Low	Low	Low	Some concerns
Bauer et al., 2009 (20)	Some concerns	Some concerns	Low	Some concerns	Some concerns	Some concerns
Alexopoulos et al., 2009 (PROSPECT) (21)	Low	Some concerns	Low	Some concerns	Low	Some concerns
Zhang et al., 2022 (22)	Low	Some concerns	Low	Some concerns	Low	Some concerns
Browning et al., 2021 (PReDicT) (23)	Low	Some concerns	Low	Some concerns	Low	Some concerns
Ell et al., 2010 (19)	Low	Some concerns	Low	Some concerns	Low	Some concerns

Risk of bias was assessed using the Cochrane Risk of Bias tool version 2 (RoB 2) for the primary outcome of each study, considering the effect of assignment to intervention (intention-to-treat). Judgements are reported as low risk, some concerns, or high risk of bias.

### 2.7 Synthesis methods

Given the substantial clinical and methodological heterogeneity across the included trials, no meta-analysis was undertaken, but narrative synthesis was performed. Consequently, no formal effect measures, data transformations, or statistical conversions were required, and a narrative synthesis was conducted with results presented in **Table 1** and in the text.

As a result of no quantitative synthesis being performed, no formal investigations of heterogeneity, sensitivity analyses, assessments of reporting bias using funnel plots or related statistical tests were conducted, as these approaches are not appropriate with fewer than ten trials (17). For the same reasons, a formal certainty of evidence assessment was not undertaken, as the small number of eligible studies and their variability in design and follow-up precluded a structured and methodologically defensible grading of certainty. No data transformations or statistical conversions were required.

## 3 Results

**Figure 1:**
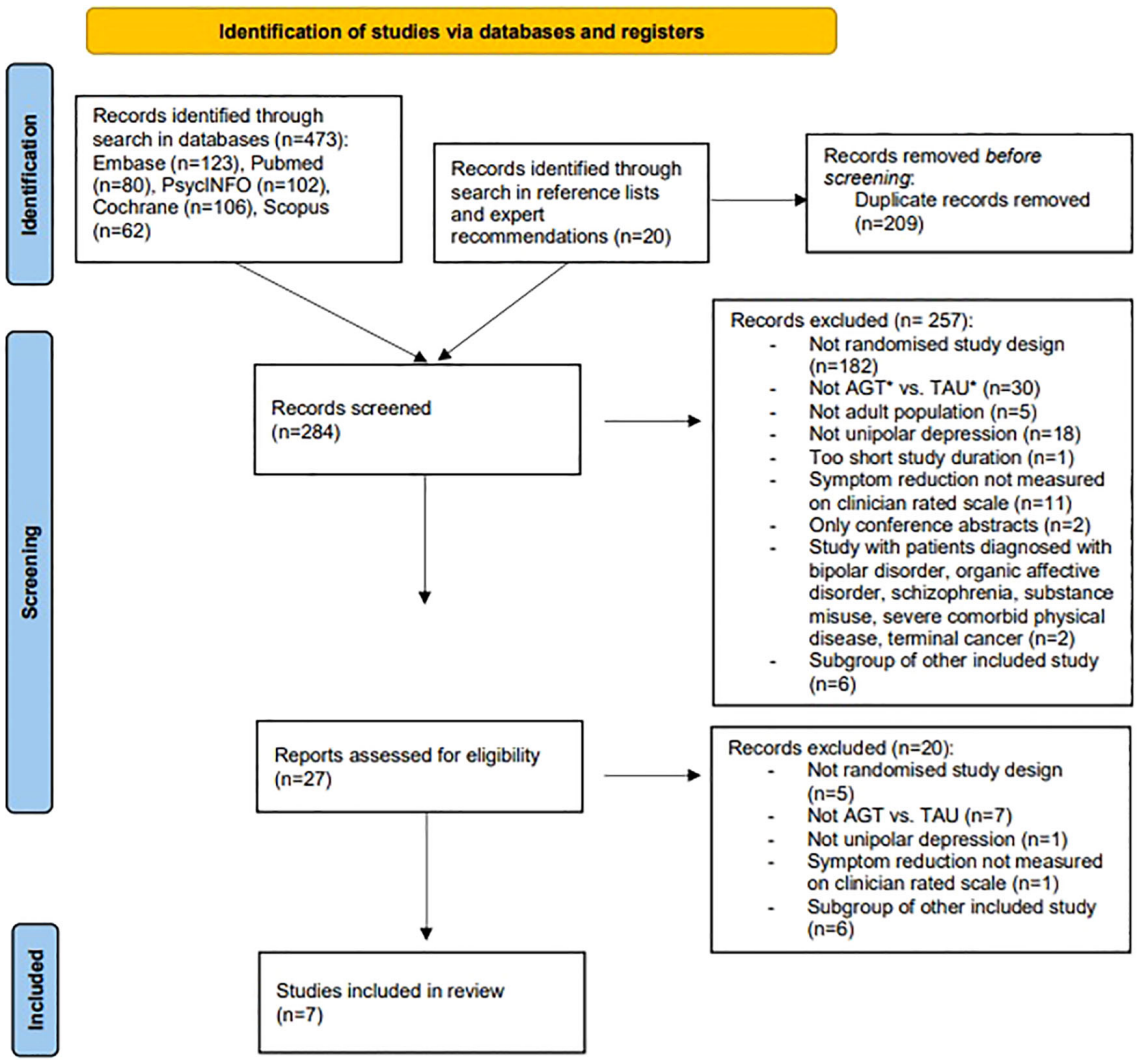
PRISMA 2020 flow diagram of the study selection process.

**Figure 1** summarizes the screening flow. Seven randomized controlled trials met the inclusion criteria, yielding a combined sample of 3,683 participants with major depressive disorder treated in inpatient units, specialist outpatient clinics, or primary-care settings (**Table 1**). Across trials, the experimental arms applied structured, measurement-based or stepped-care algorithms that prespecified dose-escalation, augmentation, or switching rules; comparators were clinician-directed treatment as usual or, in one study, also a computerized decision-support system. Study duration varied from six weeks to two years, while only one of the studies, Zhang et al. had a post-treatment follow-up after six months. Only two trials extended observation beyond 12 months, leaving longer-term durability of benefits insufficiently characterized. Trial conduct characteristics, including the unit of randomization, ITT design, dropout, and intervention fidelity, are summarized in **Table 4**.

**Table 4:** Trial conduct characteristics.

Article # and title	Unit of randomization	ITT design/analysis	Dropout	Fidelity to intervention
1. Evaluating the efficacy and moderators of algorithm-guided antidepressant treatments of major depressive disorder	Individual: block randomization within each medical center	Yes	Dropout at week 6 was 44.7% and 42.5% for two intervention groups and 34.5% for treatment as usual	Algorithm-guided recommendations were made at predefined decision points but adherence to individual algorithmic decision steps was not reported
2. The clinical effectiveness of using a predictive algorithm to guide antidepressant treatment in primary care (PReDicT): an open-label, randomised controlled trial	Individual	Yes	Approximately half of the participants did not complete the study, with similar rates in both groups (50% in the intervention group vs 48% in the usual treatment group)	Fidelity to algorithm recommendations was not quantified
3. How Effective Is Algorithm-Guided Treatment for Depressed Inpatients? Results from the Randomized Controlled Multicenter German Algorithm Project 3 Trial	Individual: randomization was performed in blocks of 10, separately for each study site	Yes	Dropout was 19% in treatment as usual and higher in intervention groups (ALGO-LA 40.7%, ALGO-DE 42.9%, ALGO-SW 42.7%, CDES 50.6%)	Fidelity was assessed indirectly. Most patients received the initial algorithm step, with remissions and dropouts occurring predominantly at step 1. ALGO pathways reduced the risk of insufficient antidepressant dosing compared with CDES, with no significant differences versus TAU
4. Measurement-Based Care Versus Standard Care for Major Depression: A Randomized Controlled Trial With Blind Raters	Individual: randomly assigned to one of groups according to a table of random numbers	Full ITT for discontinuation outcomes and modified ITT for other outcomes (baseline + ≥1 follow-up assessment)	Dropout was 27.9% for intervention group and 37.3% for treatment as usual	Treatment adherence did not differ between groups (99.8% and 99.7%)
5. Collaborative care management of major depression among low-income, predominantly Hispanic subjects with diabetes	Individual: randomization was conducted via a computer-generated random number in blocks of 10	Yes	Dropout was similar between groups at all time points. 6 months: 21.6% usual care vs 21.8% intervention 12 months: 28.4% vs 26.4% 18 months: 29.4% vs 25.4%	87.6% completed initial visit and 83.9% continued intervention. 53.9% received both psychotherapy (PST) and antidepressants (AD), 25.4% received only PST and 4.7% received only AD
6. Reducing Suicidal Ideation and Depression in Older Primary Care Patients: 24-Month Outcomes of the PROSPECT Study	Cluster: ten pairs of practices	Yes	At 24 months, 43% of intervention patients and 37% of usual care patients failed to have a research assessment	Fidelity reflects successful implementation of the collaborative care model at the practice level and intervention practices showed substantially higher rates of antidepressant treatment (approximately 85–89%) and psychotherapy use compared with usual care (approximately 49–59%)
7. Efficacy of an algorithm-guided treatment compared with treatment as usual: a randomized, controlled study of inpatients with depression	Individual: computer-generated block randomization (10 per block)	Yes	Dropout was higher in intervention group than in treatment as usual (45% vs 16%)	Fidelity was assessed indirectly via predefined algorithm deviations. Adverse drug events and physician noncompliance (protocol violations or premature discharge) were key sources, with physician noncompliance accounting for 39%

### 3.1 Remission and response

Five of the seven trials demonstrated a statistically significant advantage for AGT over treatment as usual on their primary categorical outcome (12, 18–21). Across studies, patients randomized to AGT generally performed better than those receiving TAU, with absolute differences in proportion of patients with remission ranging from 14% (21) to 45% (18). As previously mentioned, no meta-analysis was conducted. When pooling studies, the reported remission at the end of the acute treatment, the risk ratio for remission was approximately 1.6 in favor of AGT. Response rates showed a similar pattern, with the largest between-group differences observed in inpatient samples (e.g., 89.2% vs 66.2% in GAP-3 (12)).

### 3.2 Time-to-event outcomes

Three trials provided survival analyses (12, 18, 20). Median time-to-remission was shortened by 8–15 days in the German Algorithm Project (37–42 days vs 45 days) depending on which arm was compared to TAU (12) and by 5.3 weeks in the inpatient stepwise regimen study (7.0 ± 0.9 w vs 12.3 ± 1.8 w) (20). Hazard ratios for remission in algorithm arms ranged from 1.56 to 1.67 compared with TAU.

### 3.3 Continuous symptom change

Five trials showed greater mean reductions on clinician-rated depression scales in patients randomized to AGT, with between-group effect sizes in the small-to-moderate range (12, 18–21). The two studies not showing a significant effect of AGT reported advantages for algorithmic care in secondary domains such as quality of life and relapse prevention (22, 23).

### 3.4 Durability and adherence

Lastly, the two studies with follow-up longer than 2 months demonstrated sustained or widening group differences up to 24 months, including lower relapse rates in those randomized to AGT (19, 21).

AGT entailed more frequent dosage adjustments or strategy switches, yet overall attrition was comparable to TAU except in the computer-only decision-support arm of the GAP-3, where dropout reached 50% (7).

Serious adverse-event rates did not differ significantly between conditions.

## 4 Discussion

In this systematic review of seven randomized controlled trials including 3,683 adults diagnosed with depressive disorder, AGT consistently outperformed treatment as usual on several clinically important outcomes. Across most trials, AGT was associated with higher proportion of patients achieving remission and response, faster time to remission, greater improvement on clinician-rated depression scales, and comparable or better adherence, without an apparent increase in serious adverse events. Although effect sizes were generally in the small-to-moderate range, the pattern of findings converges to suggest that structured, algorithm-based approaches can meaningfully improve outcomes in major depressive disorder compared with unstructured usual care.

A key observation across studies was the benefit of AGT for achieving remission and response. Five of seven trials reported a statistically significant advantage for algorithm-guided care on their primary categorical endpoint, with absolute between-group differences in remission ranging from 14% to 45%. In addition, hazard ratios for time-to-remission in the algorithm arms (1.56–1.67) indicate a clinically relevant acceleration of recovery in several settings. The German Algorithm Project 3 (GAP-3) trial exemplifies these effects, demonstrating both higher proportion of patients with remission and shortened median time to remission in inpatients randomized to AGT compared with TAU (12). Similarly, in primary care, the PROSPECT study showed that algorithm-guided collaborative care not only improved depressive symptoms but also conferred sustained benefits over 24 months, including lower relapse rates and persistent reductions in suicidal ideation (21).

The included trials differed in how algorithms were operationalized, but they shared core elements of explicit, predefined decision rules. They included systematic symptom assessment in combination with prespecified options for dose escalation, switching, or augmentation. Most studies implemented measurement-based care, where symptom ratings at regular intervals triggered algorithmic treatment changes (18). This approach allows dynamic, iterative adaptation of treatment and may be particularly effective in preventing therapeutic inertia. Other trials relied on stepwise treatment algorithms with fixed decision points, in which treatment changes were mandated once specific response or tolerability criteria were met (20). Collaborative-care models embedded the algorithm within a broader organizational framework that included care management and psychiatric consultation (19, 21). Despite these methodological differences, the trials consistently suggest that making treatment decisions contingent on systematic assessment and explicit if–then rules improve the timeliness and adequacy of pharmacological management compared with unguided clinical judgment alone.

An important caveat is that the observed benefits of AGT are unlikely to stem from the specific content of the individual algorithms tested, but rather from the way care is structured and monitored. Across trials, the relative advantage of AGT over TAU did not clearly map onto any particular pharmacological sequence, preferred compound, or augmentation strategy. Instead, the common elements were systematic symptom monitoring, predefined decision points, and a requirement to act when insufficient improvement was documented. This suggests that it is the disciplined use of measurement-based care and clearly defined decision rules that primarily drives the improved outcomes, by counteracting therapeutic inertia and ensuring timely dose optimization, switching, or augmentation. In this sense, AGT may be viewed less as a set of competing “brands” of algorithms and more as an implementation framework that can be layered onto different evidence-based treatment options to enhance their real-world effectiveness.

Not all trials demonstrated clear superiority of AGT on their primary depression outcome. The PReDicT trial and the study by Zhang et al. yielded neutral findings on clinician-rated symptom change (22, 23). However, both reported advantages of algorithm-based approaches in secondary outcomes, including quality of life, functional outcomes, or relapse prevention. These results underscore that the benefits of AGT may not always be captured fully by short-term symptom scales alone and that broader, patient-centered outcomes can also be positively affected. They also highlight that algorithm performance may depend on setting, population, and implementation fidelity.

In the broader context of depression care, our findings align with a large body of literature suggesting that structured follow-up and measurement-based care improve treatment adequacy and clinical outcomes, particularly when coupled with clear escalation rules and organized care pathways. Many contemporary guideline frameworks emphasize systematic symptom monitoring and scheduled reassessment to reduce therapeutic inertia, and collaborative-care trials in primary care have similarly demonstrated benefits on depressive symptoms and longer-term outcomes. What this review adds beyond existing evidence is a focused synthesis of randomized trials in which treatment adjustments were explicitly governed by predefined algorithms, allowing a clearer separation of the effects of “structured, rule-based decision-making” from usual care that is often less standardized. By systematically describing algorithm features (decision thresholds, step sequences, assessment frequency, and whether decisions were clinician-led versus automated), our review supports more nuanced interpretation of where AGT may be most applicable and which components may be most important for effectiveness and feasibility.

### 4.1 Clinical implications

Overall, the evidence suggests that AGT may be a useful, pragmatic approach to address recurring challenges in depression care, particularly delayed treatment adjustments and insufficient switching or augmentation in the face of persistent non-response. By linking systematic symptom measurement to predefined decision points, AGT can help standardize follow-up and prompt timely changes when improvement is not observed, thereby reducing reliance on subjective impressions and potentially mitigating therapeutic inertia.

However, given the limited number of trials and their heterogeneity, the current findings should be interpreted as promising rather than definitive. While several studies reported higher remission/response rates and faster improvement, the magnitude and consistency of benefit likely depend on context (setting, population, and implementation fidelity). In practice, AGT may be most appropriately viewed as a structured support for clinical decision-making particularly in settings with high patient volume such as primary care or busy outpatient services rather than as a stand-alone solution. Notably, attrition was generally comparable between AGT and TAU, suggesting that structured, measurement-informed care is feasible and acceptable when implemented within routine services.

### 4.2 Limitations of evidence

Several limitations of the underlying studies must be considered when interpreting these findings. First, only seven randomized trials met our inclusion criteria, and they differed substantially in setting (inpatient vs outpatient vs primary care), population (e.g., older adults, individuals with comorbid medical illness), and the specific combinations of AGT employed (measurement-based care, stepwise algorithms, collaborative-care frameworks, or computerized decision support). Additionally, the content and intensity of TAU varied across the included studies. This clinical and methodological heterogeneity and the small number of studies precluded a robust meta-analysis and limits the precision with which pooled effect estimates can be quantified.

Second, the trials used different clinician-rated scales and outcome definitions for remission and response, which complicates direct comparison. Follow-up durations varied widely, and only two trials extended observation beyond 12 months, leaving longer-term durability of benefits insufficiently characterized. Implementation fidelity was also variably reported; in some studies, clinicians could override algorithm recommendations, and the extent to which this occurred may have diluted the observed effects of AGT.

Third, our review was limited to English-language publications and was not prospectively registered in a systematic review registry. The small number of eligible RCTs and their heterogeneity also precluded formal evaluation of small-study effects or reporting bias. These methodological constraints mean that the overall certainty of the evidence must be considered moderate at best.

Fourth, all the studies were unblinded, meaning that we could not exclude an effect of expectation and nonspecific factors in the algorithm groups, even though this was more a limitation of the individual studies than it is a limitation of our review.

### 4.3 Future directions

Future research should focus on head-to-head comparisons or factorial design of different algorithm designs (e.g., purely pharmacological algorithms vs integrated collaborative-care models; fixed stepwise algorithms vs fully measurement-based care), to determine which components are most critical for improving outcomes. Trials in more diverse populations, including treatment resistant depression, younger adults, individuals with comorbid substance use, and those treated in routine community settings, are needed to enhance generalizability. Finally, the integration of artificial intelligence, digital tools and decision support systems with AGT frameworks, building on the mixed experience with purely computerized arms, warrant careful evaluation, with particular attention to clinician engagement and safeguards around algorithm override.

## 5 Conclusion

Despite important limitations, the available randomized evidence indicates that AGT is associated with improved outcomes compared with clinician treatment as usual in adults with depressive disorders, most consistently higher rates of remission/response and, in some studies, faster time to remission and lower relapse rates without clear signals of reduced tolerability. Nevertheless, the evidence base remains limited and heterogeneous, and the certainty of conclusions is therefore moderate at best.

Taken together, these findings support further evaluation and careful, context-sensitive implementation of structured, algorithm-based and measurement-based approaches, rather than unequivocally establishing a need for universal integration into routine psychiatric and primary care practice. Additional well-designed trials and implementation studies are required to clarify which components drive benefit, in which populations and settings, and how best to embed AGT into everyday clinical workflows.

## Data Availability

The original contributions presented in the study are included in the article/**Supplementary Material**. Further inquiries can be directed to the corresponding author.

## Supplementary material

Supplementary Material 1 PRISMA 2020 checklist.

Supplementary Material 2 Search terms.

Supplementary Material 3 Protocol.
