# Supplementary material for: Algorithm-guided treatment for major depressive disorder versus treatment as usual: a systematic review
Source: Front Psychiatry. 2026 Mar 25;17:1765024. doi: 10.3389/fpsyt.2026.1765024 (PMC13056810; doi:10.3389/fpsyt.2026.1765024)
Supplement: Supplementary Material 2 — Search terms. [file Table2.docx]

PubMed

| Search | Query |
| --- | --- |
| #18 | Search: **((("Depressive Disorder"[Mesh]) OR ("Depression"[Mesh])) OR (depressi*[Title/Abstract])) AND ((((((("Algorithms"[Mesh]) OR (algorithm*[Title/Abstract])) AND (((treatment as usual*[Title/Abstract]) OR (TAU[Title/Abstract])) OR (usual treatment*[Title/Abstract]))) OR (algorithm-guided treatment*[Title/Abstract])) OR (algorithm-based treatment*[Title/Abstract])) OR (algorithm-guided therap*[Title/Abstract])) OR (algorithm-based therap*[Title/Abstract]))** Sort by: **Publication Date** |
| #17 | Search: **(((((("Algorithms"[Mesh]) OR (algorithm*[Title/Abstract])) AND (((treatment as usual*[Title/Abstract]) OR (TAU[Title/Abstract])) OR (usual treatment*[Title/Abstract]))) OR (algorithm-guided treatment*[Title/Abstract])) OR (algorithm-based treatment*[Title/Abstract])) OR (algorithm-guided therap*[Title/Abstract])) OR (algorithm-based therap*[Title/Abstract])** Sort by: **Publication Date** |
| #16 | Search: **algorithm-based therap*[Title/Abstract]** Sort by: **Publication Date** |
| #15 | Search: **algorithm-guided therap*[Title/Abstract]** Sort by: **Publication Date** |
| #14 | Search: **algorithm-based treatment*[Title/Abstract]** Sort by: **Publication Date** |
| #13 | Search: **algorithm-guided treatment*[Title/Abstract]** Sort by: **Publication Date** |
| #12 | Search: **(("Algorithms"[Mesh]) OR (algorithm*[Title/Abstract])) AND (((treatment as usual*[Title/Abstract]) OR (TAU[Title/Abstract])) OR (usual treatment*[Title/Abstract]))** Sort by: **Publication Date** |
| #11 | Search: **((treatment as usual*[Title/Abstract]) OR (TAU[Title/Abstract])) OR (usual treatment*[Title/Abstract])** Sort by: **Publication Date** |
| #10 | Search: **usual treatment*[Title/Abstract]** Sort by: **Publication Date** |
| #9 | Search: **TAU[Title/Abstract]** Sort by: **Publication Date** |
| #8 | Search: **treatment as usual*[Title/Abstract]** Sort by: **Publication Date** |
| #7 | Search: **("Algorithms"[Mesh]) OR (algorithm*[Title/Abstract])** Sort by: **Publication Date** |
| #6 | Search: **algorithm*[Title/Abstract]** Sort by: **Publication Date** |
| #5 | Search: **"Algorithms"[Mesh]** Sort by: **Publication Date** |
| #4 | Search: **(("Depressive Disorder"[Mesh]) OR ("Depression"[Mesh])) OR (depressi*[Title/Abstract])** Sort by: **Publication Date** |
| #3 | Search: **depressi*[Title/Abstract]** Sort by: **Publication Date** |
| #2 | Search: **"Depression"[Mesh]** Sort by: **Publication Date** |
| #1 | Search: **"Depressive Disorder"[Mesh]** Sort by: **Publication Date** |

Embase

| No. | Query |
| --- | --- |
| #16 | #12 AND #15 |
| #15 | #13 OR #14 |
| #14 | depressi*:ti,ab,kw |
| #13 | 'depression'/exp |
| #12 | #9 OR #10 OR #11 |
| #11 | 'algorithm-based therap*':ti,ab,kw OR 'algorithm-based treatment*':ti,ab,kw |
| #10 | 'algorithm-guided therap*':ti,ab,kw OR 'algorithm-guided treatment*':ti,ab,kw |
| #9 | #3 AND #8 |
| #8 | #4 OR #5 OR #6 OR #7 |
| #7 | 'usual treatment*':ti,ab,kw |
| #6 | tau:ti,ab,kw |
| #5 | 'treatment as usual*':ti,ab,kw |
| #4 | 'treatment as usual'/exp |
| #3 | #1 OR #2 |
| #2 | algorithm*:ti,ab,kw |
| #1 | 'algorithm'/exp |

Cochrane

| ID | Search |
| --- | --- |
| #1 | MeSH descriptor: [Depressive Disorder] explode all trees |
| #2 | MeSH descriptor: [Depression] explode all trees |
| #3 | (depressi*):ti,ab,kw |
| #4 | {OR #1-#3} |
| #5 | MeSH descriptor: [Algorithms] explode all trees |
| #6 | (algorithm*):ti,ab,kw |
| #7 | #5 or #6 |
| #8 | (treatment* NEAR/2 usual*):ti,ab,kw |
| #9 | (TAU):ti,ab,kw |
| #10 | #8 OR #9 |
| #11 | #7 AND #10 |
| #12 | (algorithm-based NEAR/1 (treatment* OR therap*)):ti,ab,kw |
| #13 | (algorithm-guided NEAR/1 (treatment* OR therap*)):ti,ab,kw |
| #14 | #11 OR #12 OR #13 |
| #15 | #4 AND #14 |

PsycINFO

| # | Query |
| --- | --- |
| 1 | ((**IndexTermsFilt**: ("Major Depression") *OR* **IndexTermsFilt**: ("Depression (Emotion)")) *OR* (**title**: (depressi*)) *OR* (**abstract**: (depressi*)) *OR* (**Keywords**: (depressi*))) *AND* ((((Title:(algorithm-based NEAR/1) (Title:(treatment*) OR Title:(therap*))) OR (Title:(algorithm-guided NEAR/1) (Title:(treatment*) OR Title:(therap*))) OR (Abstract:(algorithm-based NEAR/1) (Abstract:(treatment*) OR Abstract:(therap*))) OR (Abstract:(algorithm-guided NEAR/1) (Abstract:(treatment*) OR Abstract:(therap*))) OR (Subject:(algorithm-based NEAR/1) (Subject:(treatment*) OR Subject:(therap*))) OR (Subject:(algorithm-guided NEAR/1) (Subject:(treatment*) OR Subject:(therap*))))) OR (((((title:(algorithm*)))) OR (((abstract:(algorithm*)))) OR (((Subject:(algorithm*)))) OR (((IndexTermsFilt:("Algorithms"))))) AND ((((title:(treatment* NEAR/2 usual*)))) OR (((abstract:(treatment* NEAR/2 usual*)))) OR (((Subject:(treatment* NEAR/2 usual*))))))) *AND* **Publication Type**: Peer Reviewed Journal  *Search Databases:*APA PsycInfo, APA PsycArticles, APA PsycTests, APA PsycTherapy |
| 2 | ((**IndexTermsFilt**: ("Major Depression") *OR* **IndexTermsFilt**: ("Depression (Emotion)")) *OR* (**title**: (depressi*)) *OR* (**abstract**: (depressi*)) *OR* (**Keywords**: (depressi*))) *AND* ((((Title:(algorithm-based NEAR/1) (Title:(treatment*) OR Title:(therap*))) OR (Title:(algorithm-guided NEAR/1) (Title:(treatment*) OR Title:(therap*))) OR (Abstract:(algorithm-based NEAR/1) (Abstract:(treatment*) OR Abstract:(therap*))) OR (Abstract:(algorithm-guided NEAR/1) (Abstract:(treatment*) OR Abstract:(therap*))) OR (Subject:(algorithm-based NEAR/1) (Subject:(treatment*) OR Subject:(therap*))) OR (Subject:(algorithm-guided NEAR/1) (Subject:(treatment*) OR Subject:(therap*))))) OR (((((title:(algorithm*)))) OR (((abstract:(algorithm*)))) OR (((Subject:(algorithm*)))) OR (((IndexTermsFilt:("Algorithms"))))) AND ((((title:(treatment* NEAR/2 usual*)))) OR (((abstract:(treatment* NEAR/2 usual*)))) OR (((Subject:(treatment* NEAR/2 usual*)))))))  Search Databases: APA PsycInfo, APA PsycArticles, APA PsycTests, APA PsycTherapy |
| 3 | ((Title:(algorithm-based NEAR/1) (Title:(treatment*) OR Title:(therap*))) OR (Title:(algorithm-guided NEAR/1) (Title:(treatment*) OR Title:(therap*))) OR (Abstract:(algorithm-based NEAR/1) (Abstract:(treatment*) OR Abstract:(therap*))) OR (Abstract:(algorithm-guided NEAR/1) (Abstract:(treatment*) OR Abstract:(therap*))) OR (Subject:(algorithm-based NEAR/1) (Subject:(treatment*) OR Subject:(therap*))) OR (Subject:(algorithm-guided NEAR/1) (Subject:(treatment*) OR Subject:(therap*)))) *OR* ((((**title**: (algorithm*))) *OR* ((**abstract**: (algorithm*))) *OR* ((**Keywords**: (algorithm*))) *OR* ((**IndexTermsFilt**: ("Algorithms")))) *AND* (((**title**: (treatment* NEAR/2 usual*))) *OR* ((**abstract**: (treatment* NEAR/2 usual*))) *OR* ((**Keywords**: (treatment* NEAR/2 usual*)))))  Search Databases: APA PsycInfo, APA PsycArticles, APA PsycTests, APA PsycTherapy |
| 4 | ((**title**: (algorithm*)) *OR* (**abstract**: (algorithm*)) *OR* (**Keywords**: (algorithm*)) *OR* (**IndexTermsFilt**: ("Algorithms"))) *AND* ((**title**: (treatment* NEAR/2 usual*)) *OR* (**abstract**: (treatment* NEAR/2 usual*)) *OR* (**Keywords**: (treatment* NEAR/2 usual*)))  Search Databases: APA PsycInfo, APA PsycArticles, APA PsycTests, APA PsycTherapy |
| 5 | (**Title**: treatment* NEAR/2 usual*) *OR* (**Abstract**: treatment* NEAR/2 usual*) *OR* (**Keywords**: treatment* NEAR/2 usual*)  Search Databases: APA PsycInfo, APA PsycArticles, APA PsycTests, APA PsycTherapy |
| 6 | (**Title**: algorithm-based NEAR/1 (**Title**: treatment* *OR* **Title**: therap*))  *OR* (**Title**: algorithm-guided NEAR/1 (**Title**: treatment* *OR* **Title**: therap*))  *OR* (**Abstract**: algorithm-based NEAR/1 (**Abstract**: treatment* *OR*  **Abstract**: therap*)) *OR* (**Abstract**: algorithm-guided NEAR/1 (**Abstract**: treatment*  *OR* **Abstract**: therap*)) *OR* (**Keywords**: algorithm-based NEAR/1 (**Keywords**: treatment* *OR* **Keywords**: therap*))  *OR* (**Keywords**: algorithm-guided NEAR/1 (**Keywords**: treatment* *OR*  **Keywords**: therap*))  Search Databases: APA PsycInfo, APA PsycArticles, APA PsycTests, APA PsycTherapy |
| 7 | **Title**: algorithm* *OR* **Abstract**: algorithm* *OR* **Keywords**: algorithm* *OR* **Index Terms**: {Algorithms}  Search Databases: APA PsycInfo, APA PsycArticles, APA PsycTests, APA PsycTherapy |
| 8 | **Index Terms**: {Major Depression} OR {Depression (Emotion)} *OR* **Title**: depressi* *OR* **Abstract**: depressi* *OR* **Keywords**: depressi*  Search Databases: APA PsycInfo, APA PsycArticles, APA PsycTests, APA PsycTherapy |

Scopus

| # | Query |
| --- | --- |
| 6 | ( TITLE-ABS-KEY ( depressi* ) ) AND ( ( TITLE-ABS-KEY ( algorithm-guided W/2 ( treatment* OR therap* ) ) ) OR ( TITLE-ABS-KEY ( algorithm-based W/2 ( treatment* OR therap* ) ) ) ) AND ( EXCLUDE ( DOCTYPE , "cp" ) OR EXCLUDE ( DOCTYPE , "ch" ) ) |
| 5 | ( TITLE-ABS-KEY ( depressi* ) ) AND ( ( TITLE-ABS-KEY ( algorithm-guided W/2 ( treatment* OR therap* ) ) ) OR ( TITLE-ABS-KEY ( algorithm-based W/2 ( treatment* OR therap* ) ) ) ) |
| 4 | ( TITLE-ABS-KEY ( algorithm-guided W/2 ( treatment* OR therap* ) ) ) OR ( TITLE-ABS-KEY ( algorithm-based W/2 ( treatment* OR therap* ) ) ) |
| 3 | TITLE-ABS-KEY ( algorithm-based W/2 ( treatment* OR therap* ) ) |
| 2 | TITLE-ABS-KEY ( algorithm-guided W/2 ( treatment* OR therap* ) ) |
| 1 | TITLE-ABS-KEY ( depressi* ) |

Supplementary material 2. Search terms
