# Supplementary material for: Algorithm-guided treatment for major depressive disorder versus treatment as usual: a systematic review
Source: Front Psychiatry. 2026 Mar 25;17:1765024. doi: 10.3389/fpsyt.2026.1765024 (PMC13056810; doi:10.3389/fpsyt.2026.1765024)
Supplement: Supplementary Material 3 — Protocol. [file Table3.docx]

Algorithm-guided treatment for major depressive disorder versus treatment as usual: a systematic review

Protocol

**Introduction**

Depressive disorders are one of the leading causes of burden worldwide and were placed second and thirteenth among the top 25 leading causes of years lived with disabilities (YLDs) and disability-adjusted life-years (DALYs), respectively (1). Even with a wide range of therapy choices, chronic and treatment refractory depression still presents a significant challenge.
It has been shown that about 33% of patients with major depressive disorder (MDD) achieved remission after the first treatment step (2). Among those who did not remit, an additional 20% remitted after the second step, bringing the cumulative remission rate to approximately 53%. A further 10% of patients remitted after the third step, and 7% after the fourth, resulting in a total cumulative remission rate of around 70% after four successive treatment trials. These findings highlight that with each additional failed trial, the likelihood of remission declines substantially, and that patients who do not respond early are increasingly at risk of developing chronic or treatment-resistant depression. This underlines the importance of early identification of non-response and timely adjustment of treatment strategies and underscoring the importance of promptly adjusting ineffective treatment strategies to optimize outcomes (3).

One possible strategy for addressing these challenges is algorithm-guided treatment (AGT). The essential elements of AGTs are which treatments to use, how to implement each treatment and in what order to implement the different treatments (4). Furthermore, AGTs also define critical decision points during treatment at which the results of a certain treatment are assessed and based on this assessment recommend specific treatment revisions according to preset “if-then rules.” Development of systematic and diligent approach should enhance patient outcomes, reduce treatment resistance, increase the quality of care, and potentially decrease direct and indirect costs of health care (5). By using algorithms, clinicians are provided with a clear framework for decision-making, helping them determine when and how to adjust treatment based on the patient's response. It has been shown that, in comparison to treatment as usual, algorithm-guided treatments and collaborative care approaches are associated with greater and more rapid reductions in symptoms, higher patient satisfaction, and improved overall quality of life (6).

Several studies have been conducted investigating the role of systematic, stepwise, drug treatment protocols and regimes in individuals diagnosed with depression. Some of these studies, such as Sequenced Treatment Alternatives to Relieve Depression (STAR*D) (2), compared different treatment strategies with each other, while for instance German Algorithm Project 3 Trial (GAP3) compared algorithm-guided treatments with treatment as usual (7). The Texas Medication Algorithm Project chose to do cluster randomization in which treatment arms were allocated by clinics (8).

**Objectives**

The primary objective of this systematic review is to evaluate the clinical effectiveness of algorithm-guided treatment compared with treatment as usual in adults (≥18 years) with major depressive disorder. Our goal was to assess whether AGT leads to superior outcomes in terms of remission rates, response rates, time to remission, and overall treatment adherence. The findings of this review are intended to inform clinical decision-making and support the potential implementation of algorithm-based approaches in routine psychiatric care.

**Inclusion criteria**

1. Only randomized controlled trials will be included

2. Studies must include adult participants (≥18 years) diagnosed with a major depressive disorder. Studies including comorbid psychiatric or somatic conditions will be eligible, except for the exclusion criteria listed below

3. Intervention: Algorithm-guided treatment

4. Comparator: Treatment as usual

5. Studies must report symptom change using a clinician-rated depression scale

6. Included trials must have a minimum duration of four weeks

7. Only studies published in English will be included

**Exclusion criteria**

1. Studies including patients diagnosed with bipolar disorder, organic affective disorders, schizophrenia, substance misuse, severe comorbid physical disease, or terminal cancer

2. Trials published only as conference abstracts

**Information sources**

The following electronic databases will be searched: PubMed, Scopus, Embase, PsycINFO and Cochrane Library

**Search strategy**

Core search terms will include: (algorithm-based treatment OR algorithm-guided treatment) AND (treatment as usual) AND (depressi*)
The original electronic search strategy will be documented prior to review completion.

**Study selection**

Two reviewers will independently screen titles and abstracts for eligibility. Full-text articles will be retrieved for potentially eligible studies and assessed independently by the same reviewers. Disagreements will be resolved through discussion or by consultation with a third reviewer.

**Data extraction**

Data will be extracted independently by two reviewers using a pre-specified extraction form. Extracted data will include study identification (title, authors, year), participant characteristics (sample size, age, sex), trial duration and outcomes.

**Ethics and dissemination**

This review will synthesize data from published studies and will not involve collection of new data. Ethical approval is therefore not required.

**References**

1. Mental GBD, Collaborators D. Global, regional, and national burden of 12 mental disorders in 204 countries and territories, 1990–2019: a systematic analysis for the Global Burden of Disease Study 2019. The Lancet Psychiatry [Internet]. 2022;9(2):137–50. Available from: http://dx.doi.org/10.1016/S2215-0366(21)00395-3

2. John Rush A, Trivedi MH, Wisniewski SR, Nierenberg AA, Stewart JW, Warden D, et al. Acute and Longer-Term Outcomes in Depressed Outpatients Requiring One or Several Treatment Steps: A STAR*D Report [Internet]. Vol. 163, Am J Psychiatry. 2006. Available from: www.star-d.org

3. Adli M, Rush AJ, Möller HJ, Bauer M. Algorithms for Optimizing the Treatment of Depression: Making the Right Decision at the Right Time. Pharmacopsychiatry. 2003;36(SUPPL. 3).

4. Yoshino A, Sawamura T, Kobayashi N, Kurauchi S, Matsumoto A, Nomura S. Algorithm-guided treatment versus treatment as usual for major depression: Regular Article. Psychiatry Clin Neurosci. 2009;63(5):652–7.

5. Bauer M, Rush AJ, Ricken R, Pilhatsch M, Adli M, Bauer M, et al. Algorithms For Treatment of Major Depressive Disorder : Efficacy and Cost-Effectiveness Authors What contrasts practice treatment guidelines from algorithms ? 2018;

6. Adli M, Bauer M, Rush AJ. Algorithms and Collaborative-care Systems for Depression: Are They Effective and Why?. A Systematic Review. Biol Psychiatry. 2006;59(11):1029–38.

7. Adli M, Wiethoff K, Baghai TC, Fisher R, Seemüller F, Laakmann G, et al. How effective is algorithm-guided treatment for depressed inpatients? results from the randomized controlled multicenter German algorithm project 3 trial. Int J Neuropsychopharmacol. 2017;20(9):721–30.

8. Kashner TM, Carmody TJ, Suppes T, Rush AJ, Crismon ML, Miller AL, et al. Catching up on health outcomes: The Texas medication algorithm project. Health Serv Res. 2003;38(1 I):311–31.
